# Factors associated with public attitudes towards persons with disabilities: a systematic review

**DOI:** 10.1186/s12889-021-11139-3

**Published:** 2021-06-03

**Authors:** Ziru Wang, Xiaoli Xu, Qiong Han, Yan Chen, Jiayao Jiang, Guo-Xin Ni

**Affiliations:** 1 School of Sports Medicine and Rehabilitation, Beijing Sport University, Beijing, 10084 China; 2 Department of Rehabilitation Medicine, The First Affiliated Hospital of Fujian Medical University, Fuzhou, China

**Keywords:** Factors, Attitudes, Disabilities, Systematic review

## Abstract

**Objective:**

The aim of this review is to identify and summarize factors that are associated with public attitudes towards people with various disabilities systematically.

**Methods:**

An electronic search of three databases was performed (Medline, EMBASE and Cochrane) covering the period from 1950 to present. A comprehensive search strategy was developed and the lists of citations were screened for potential eligible studies. Only quantitative studies using valid measurements were included, and the methodological quality of included studies was appraised based on three criteria (sample, measurement, analysis) by two independent reviewers.

**Results:**

The initial electronic search yielded 995 articles after duplicates removed, and 27 studies met the eligibility criteria were included in the study**.** Three categories of the factors were found to be associated with the public attitudes, which are related to the attitude provider, disabled people, and society respectively. Specifically, the more people know about disabilities, the more likely they were to have positive attitude; and the frequency and quality of the contact with the disabled are also proved to be influential to the attitudes. Meanwhile, the type of disability is also closely correlated to the public’s attitude towards the disabilities.

**Conclusion:**

People’s knowledge of the disability and their contact with individuals with disabilities are the main influential factors in public attitudes towards persons with disabilities.

## Key messages

- This study provides a comprehensive overview of attitudes toward the general disabled, and it fills the gap in the current knowledge through a wider focus that goes beyond a specific aspect (e.g., a specified type of disability or a specific domain of the attitude).
- There are three categories of factors that found to be associated with the public attitudes, which are related to the attitude provider, disabled people, and society respectively.
- People’s knowledge of the disability, and the quality and frequency of their contact with the disabled are the main factors that influence public attitude towards persons with various disabilities.

## Background

Disability has become a natural part of the global human condition across various areas, due to the growing aging population, and the changes of demographics of societies [1]. According to the World Health Organization [2], disability, which is extremely diverse, results from the interaction between individuals with a health condition and personal and environmental factors. Indeed, there are more than one billion people (15% of the world’s population) experiencing different types of disability, which also poses a serious medical and social burden in the world [3]. Therefore, it evokes the public to consider appropriate strategies to include and support people with disabilities. Recent years, many countries have started to develop social and rehabilitation plans to overcome the burden and enhance the well-being of people with mental and physical disabilities in their community [4].

Nothing is more essential to the well-being of people with disabilities than acceptance and support given by the public [5]. As by Helen Keller, a famous disabled writer with disabilities, “the chief handicap of the blind is not blindness, but the attitude of seeing people towards them”. Attitudes toward the disability involve multidimensional evaluation of people, which can be either positive or negative, or comprised of both [6]. A number of studies have addressed the impacts of different attitudes, for example, positive social attitudes could facilitate inclusion and facilitate acceptance by family, friends, and employers [7], while negative attitudes may lead to low expectations, discrimination, and marginalization [8]. To be more specific, evidence showed that negative attitudes of the healthcare professionals have been indicated as a barrier for the participation of individuals with disabilities in several demands such as physical activity, fitness, and education settings [9]. Given this global situation and the importance of attitude, the public must be urged to rethink and promote their attitudes towards people with disabilities, in order to build a more inclusive society.

Evidence shows that social inclusion, community participation and the empowerment of people with disabilities, are central concepts guiding current policies and services provided around the world [10]. Public attitudes towards disabled people not only affect their integration into the community and access to public services [11, 12] but also influence their daily lives and social participation [7, 11], such as employment [13]. As recognized by several studies [14, 15], attitudes can be formed by people’s past and present experience, indicating that a variety of factors could mediate and impact public attitudes toward disabled people. The concept of attitude is multidimensional [16, 17], but public attitudes towards people with various disabilities have not been addressed in current available studies. It is therefore necessary to identify the influential factors and determine if the association between those factors and the public attitudes exits. This could provide insights into appropriate measures to not only promote the positive attitudes [15], but also modify the negative attitudes [4]. An overview of potential influential factors, both hindering and facilitating, could provide information for health professionals, educators, and policy maker to develop effective interventions and decisions.

Therefore, this study aims to systematically collect, identify, and evaluate factors that associated with public attitudes towards people with disabilities, so as to provide a basis for the further study targeting this area.

## Methods

### Search strategy and eligibility criteria

In March 2020, we searched the following electronic databases, starting from their dates if inception: Medline (Ovid), EMBASE and Cochrane. Articles from 1950 to the present were searched. A comprehensive search strategy was developed on three major themes- (a) attitude, (b) factor and (c) disability with individual search terms for each database, such as: [“Attitud*” OR “belief” OR “ageis*” OR “agis*” OR “discriminat*” OR “prejudic*” OR “stereotyp*” OR “stigma”] AND [“physically challenged OR handicap* OR disabled OR disabilities OR disability OR impairment OR disorder]. In addition, we screened the citation lists of included and relevant papers for potentially eligible studies. Studies retrieved from the initial searches were screened using the following inclusion criteria: (1) study aim is to understand influencing factors associated with public attitudes, (2) study outcome is related to the attitude toward people with disabilities, and (3) study design is cross-sectional or cohort. Exclusion criteria were as follows: (1) qualitative design, (2) study evaluating an intervention, and (3) report language is non-English.

Endnote was used to list all literatures retrieved in the database and to check for duplicates. Two researchers independently screened the titles and abstracts of the remaining non-duplicates to exclude the irrelevant literatures. In case of divergence, the third senior researcher evaluated the duplicates and the uncertainties. After removing the unrelated literatures second time, two researchers read the full text respectively, the remaining records were selected in accordance with the inclusion and exclusion criteria.

### Quality assessment and data synthesis

Only quantitative studies using valid measurements were included in this review, and correlation analyses or difference testing results were appraised for each study. The methodological quality of the 27 included studies was rated by evaluating three key criteria based on the McMaster Critical Review Form for quantitative research. The criterion ‘sample’ examined whether or not selection bias was reduced, the sample size was appropriate for the study design and research objective, and the characteristics of the participants were fully described. The criterion ‘measurement’ examined whether or not measurement bias was controlled for the: subject, observer, procedure, and instrument. The criterion ‘analyses’ examined whether or not the analyses tests were matched to the research question, the outcome measurement scale, and the nature (e.g., category and numbers) of the outcome and exposure variables. Each criterion was scored with one star (no evidence of meeting the requirement), or two stars (report but unclear, insufficient evidence), or three stars (has evidence meeting the requirement). Two reviewers independently performed the methodological quality assessment. Any discrepancies between the two reviewers were discussed until a consensus was reached. If a consensus could not be reached, agreement was obtained through discussions with a third reviewer. We did not conduct any meta-analysis given the heterogeneity of the reporting instruments employed.

After systematic collection and evaluation, synthesis of original literature could: describe the extent to which current factors influence public’s attitudes toward the disabled, identify the existing problems and gaps, and provide implications for improving the attitudes toward the disabled. The included studies were independently reviewed and summarized by two reviewers. Any discrepancies were resolved through discussion. Descriptive data, including literature authors, publication dates, subjects, types of disability, tools and results, were extracted from the studies using a uniform table. The findings were then categorized to summarize the state of the studies for different associated variables.

## Results

**Fig. 1:**
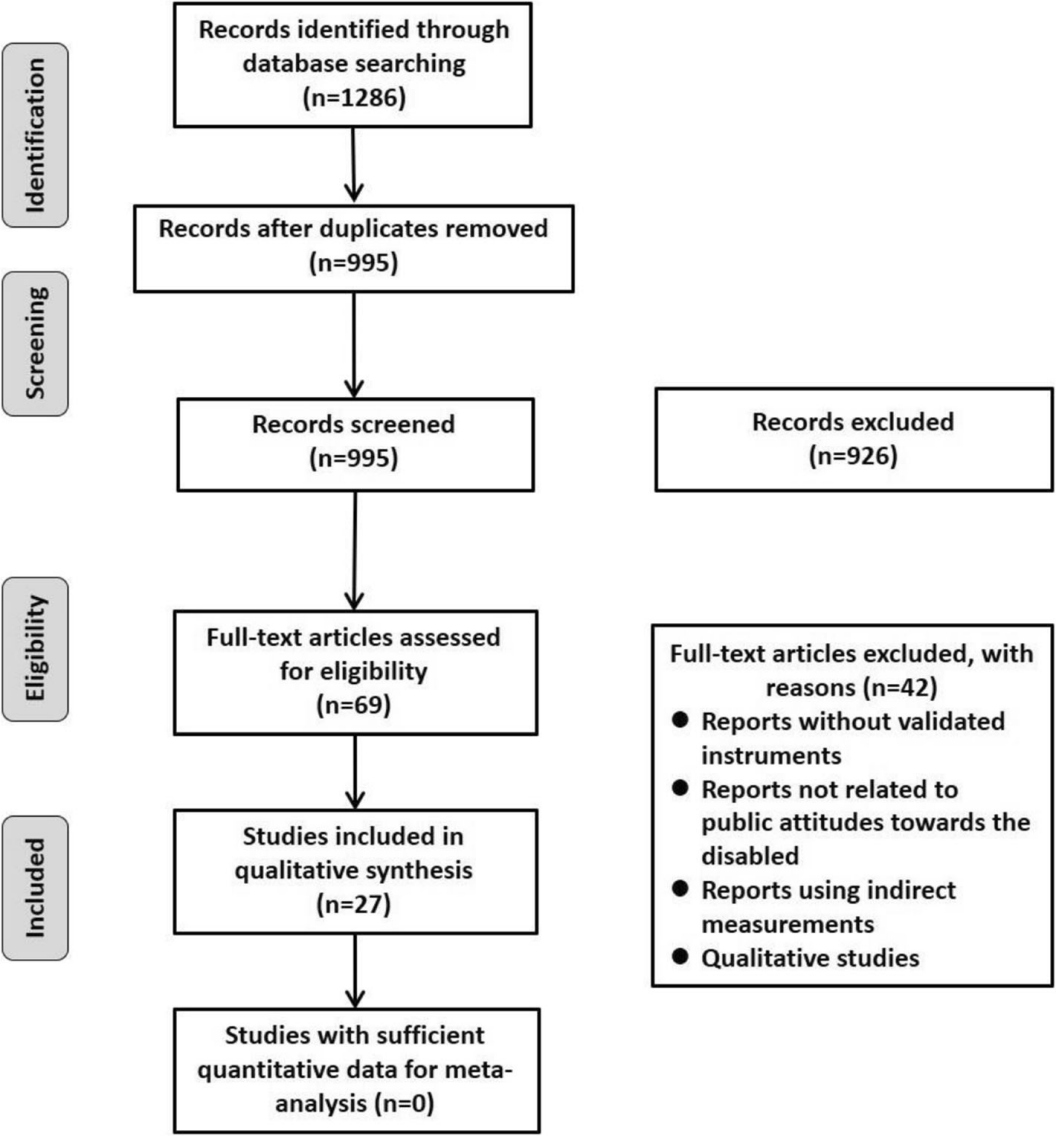
Study selection process

Initial screening of the 1286 search results from the main search removed 291 duplicates, and the remaining 995 records were screened. Nine hundred twenty-six articles did not address the general topic in title or abstract, and the remaining 69 articles were scanned full text for eligibility. Following this, 42 articles were removed because they did not measure attitudes using validated instruments, did not address public attitudes towards disabled people, or did not use direct measurements. After the initial electronic search and the manual search of the reference according to the inclusion and exclusion criteria, a total of 27 studies met the eligibility criteria for the final review. A flow diagram of literature search and selection process was presented in Fig. 1. The risk of bias of this study is low and we tried to avoid the selection bias by critically apprising each included one, while publication bias may exist since statistically significant studies were more likely to be published.

Table 1 provides the methodological quality rating results for the included studies. Only one study [18] scored the maximum rating of three stars for all three criteria, while none of the included studies scored the rating of one star for any criteria. Seven studies reported the evidence that meets the measurement criteria, but the others haven’t justified clearly enough about the bias control, or the psychometric properties of the instrument. Eleven studies [14, 18–27] scored three stars for “analysis”, which indicated the provision of sufficient evidence and the selection of the appropriate analysis strategy for the given outcome measuring scale and have provided enough evidence, whereas the others rated two stars. The participants in most of the included studies were in a younger age range (e.g., students), which might have caused a selection bias.

**Table 1:** Quality of the included studies

Ref.	Sample	Measurement	Analyses
10	✭✭	✭✭	✭✭
14	✭✭✭	✭✭	✭✭✭
21	✭✭	✭✭	✭✭✭
22	✭✭	✭✭	✭✭
18	✭✭	✭✭	✭✭
20	✭✭✭	✭✭	✭✭✭
23	✭✭	✭✭	✭✭
19	✭✭	✭✭	✭✭
24	✭✭	✭✭✭	✭✭
25	✭✭	✭✭	✭✭✭
26	✭✭✭	✭✭	✭✭✭
27	✭✭	✭✭	✭✭✭
28	✭✭	✭✭	✭✭
35	✭✭✭	✭✭	✭✭✭
36	✭✭	✭✭✭	✭✭
32	✭✭	✭✭	✭✭
39	✭✭	✭✭✭	✭✭
34	✭✭✭	✭✭	✭✭
31	✭✭	✭✭	✭✭
33	✭✭	✭✭✭	✭✭
29	✭✭	✭✭	✭✭
40	✭✭✭	✭✭✭	✭✭✭
41	✭✭	✭✭✭	✭✭
30	✭✭	✭✭✭	✭✭
42	✭✭	✭✭	✭✭✭
37	✭✭✭	✭✭	✭✭✭
38	✭✭	✭✭	✭✭✭

✭No evidence of study meeting criteria; ✭✭Some evidence of study meeting criteria or unclear reporting; ✭✭✭Evidence of study meeting criteria

The included studies revealed that a variety of factors were associated with public attitudes towards persons with disabilities. These factors were divided into three categories, namely, factors related to the provider, factors related to the disabled, and factors related to the society.

### Factors related to the attitude holder

Relationships between variables related to the provider and their attitudes toward people with disabilities across 25 studies are demonstrated in Table 2.

**Table 2:** Variables related to provider

Factors	Ref.	Instruments	Study population and setting	Results
Gender	[10]	ATTID	Participants: 1605adults Setting: Québec, Canada	While men have more negative attitudes regarding discomfort, women have more negative attitudes to the knowledge about competence and rights
	[20]	CLAS-MR (Form A & B)	Participants: 452 adults Setting: Karachi, Pakistan	Females hold more positive attitudes toward individuals with intellectual disability
	[28]	TATDP	Participants: University students (582 from Medical School, 224 from School of Nursing) Setting: Ege University, Turkey	Females have better attitude towards the disabled people than males
	[29]	CATCHs; MAS	Participants: 200 high school and 144 university students Setting: Nijmegen, Netherlands	Girls have more positive attitude towards the disabled
	[19]	ATDP (Form B)	Participants: 297 medical and dental students and healthcare professionals Setting: San Francisco, United States	Compared with men, women have more positive attitude towards people with physical disabilities
	[30]	ATDP (Form B)	Participants: 634 college students, and 234 healthcare professionals Setting: Tel Aviv University, Israel	Gender is not related to attitudes among students
	[31]	A specially designed attitude questionnaire	Participants: 129 individuals Setting: Pennsylvania, United state	Women have more positive attitude towards the disabled than men
	[32]	ATDP (Form A)	Participants: 197 clinical physiotherapy students Setting: Three Universities in Nigeria	Gender has no influence on attitude
Age	[10]	ATTID	Participants:1605 participants Setting: Québec, Canada	More positive attitudes are revealed among younger participants.
	[14]	ADS	Participants: 2912 people with disability, 507 caregivers, and 354 members of the public Setting: Guangzhou, China	Older people have more negative effects on attitude towards disability
	[20]	CLAS-MR (Form A & B)	Participants: 452 Pakistani nationals Setting: Karachi, Pakistan	Younger individuals have more negative attitudes towards the disabled
	[29]	CATCHs; MAS	Participants: 200 high school and 144 university students Setting: Nijmegen, Netherlands	The older the respondents, the more positive their attitudes towards the disabled
	[19]	ATDP (Form B)	Participants: 297 medical and dental students, and healthcare professionals Setting: San Francisco, United state	Age was not significantly correlated with ATDP scores, and would have no effect on attitudes.
	[31]	A specially designed attitude questionnaire	Participants: 129 individuals Setting: Pennsylvania, United States	Younger adults generally voice more favorable attitudes than older adults
	[32]	ATDP (Form A)	Participants: 197 clinical physiotherapy students Setting: Three universities in Nigeria	Older students have better attitudes towards the disabled
	[21]	MRAI-R	Participants:135 participants Setting: Taiwan, China	Old people tend to have more positive attitude to the disabled
	[22]	ATDP (Form O)	Participants: 587 undergraduate nursing students Setting: Three cities in Turkey	People between 18 and 21 years old are more positive towards the disabled than people aged 22 and over
	[23]	ATDP (Form A);SADP;CLAS-MR	Participants: 78 nursing students and 43 non-nursing peers Setting: Netherlands	Older age is a marginally statistically significant predictor of a more positive attitude to physically disabled persons by the ATDP-A, but not the SADP
	[33]	ATDP (Form O)	Participants: 67 baccalaureate nursing students Setting: University in the Midwest, United States	Age fails to contribute significantly to the change in nursing students’ attitudes
Education	[10]	ATTID	Participants: 1605 adults Setting: Québec, Canada	More positive attitudes are revealed among better educated participants
	[20]	CLAS-MR (Form A &B)	Participants: 452 Pakistani nationals Setting: Karachi, Pakistan	Well-educated Pakistanis are more positive about people with intellectual disabilities
	[33]	ATDP (Form O)	Participants: 67 baccalaureate nursing students Setting: United States	Junior and senior students show more positive attitudes than sophomore students towards the disabled
	[22]	ATDP (Form A)	Participants:197 clinical physiotherapy students Setting: Three Universities in Nigeria	Students of the University of Maiduguri had more positive attitude compared to students of the University of Ibadan and Nnamdi Azikiwe University
Contact	[10]	ATTID	Participants: 1605 adults Setting: Québec, Canada	The more frequent the contact, the more positive the attitudes
	[14]	ADS	Participants:2912 people with disability, 507 caregivers, and 354 members of the public setting: Guangzhou, China	The longer caregivers cared for disabled people, the more negative attitudes towards the disabled people
	[28]	TATDP	Participants: University students (582 from Medical School, 224 from School of Nursing) Setting: Ege University, Turkey	Those who were previously in close contact with disabled people have significantly better attitude than those who were not.
	[19]	ATDP (Form B)	Number: 297 medical and dental students and healthcare professionals Setting: San Francisco, United State	The frequent contact individuals have better attitude towards the disabled
	[21]	MRAI-R	Participants:135 healthy participants Setting: Taiwan, China	The longer they worked with colleagues with disabilities, the more positive their mood was
	[22]	ATDP (Form O)	Participants: 587 undergraduate nursing students Setting: Three cities in Turkey	Whether students had experience of contacting with disabled in clinical practice, there was no statistically significant difference in students’ attitude
	[34]	SADP	Participants:338 Chinese students in three secondary schools Setting: Hong Kong, China	Students who had the least contact with the disabled are more optimistic and concerned about the human rights situation of the disabled and have fewer misunderstandings about the disabled.
	[35]	the Interaction with Disabled Persons scale; the Community Living Attitudes scale; and the Barriers to Exercise scale	Participants: 16 students and 16 young adults with Down syndrome Setting: Australia	Contact with young adults with disabilities can lead to positive changes in students’ attitudes towards disability
	[36]	GNAT	Participants:550adults Setting: United States	Higher quality of contact predicted stronger positive implicit attitudes toward intellectual and developmental disability; however quantity of contact was related to higher levels of explicit prejudice.
	[37]	The Disability Questionnaire	Participants:142 employers Setting:Colorado Springs, United States	Having a high level of experience working with disabled employees can generate positive employer attitudes
	[38]	Students’ Attitudes toward People with a Disability Scale	Participants:406 students at a mainstream secondary school Setting: Hong Kong	Students having social contact and participating educational programs have a higher positive change in their attitudes.
Familiarity	[29]	CATCHs; MAS	Participants: 200 high school and 144 university students Setting: Nijmegen, Netherlands	Being familiarity with a disabled person has a significant positive effect on attitudes
	[20]	CLAS-MR (Form A & B)	Participants:452 Pakistani nationals Setting: Karachi, Pakistan	Participants who reported having a friend or relative with a disability have significantly different attitudes than individuals without a friend or relative with a disability
	[23]	ATDP (Form A)	Participants: 78 nursing students and 43 non-nursing peers Setting: Netherlands	An important additional predictor of a more positive attitude about physically disabled people was having a relative or friend with a physical disability, but this association was not apparent in attitudes towards intellectually disabled persons
	[33]	ATDP (Form O)	Participants: 67 baccalaureate nursing students Setting: United States	There were no significant differences in attitudes toward people with disabilities based on having a family member or friend with a disability or being in frequent personal contact with a disabled individual.
	[39]	DSDS	Participants: 402 entry-level occupational therapists Setting: United States	Respondents who exhibited a greater amount of nonclinical contact with persons with disabilities would exhibit more positive attitudes toward these persons
	[24]	The Interaction with Disabled Persons’ Scale	Participants:2299 students from 37physiotherapy and 24 occupational therapy schools Setting: United Kingdom	Students who have found a family member with a disability or who has an informal social connection with a person with a disability are more positive than those who do not.
	[40]	ATDP (Form O)	Participant:166 college students Setting: United States	Previous working experiences with people with disabilities have a greater positive attitude than those who do not work with people with disabilities,
	[25]	CATCH	Participant: 357 elementary school male students (grades 3–6) Setting: Riyadh city, Saudi Arabia	Participants from schools that included students with intellectual disabilities had more positive attitudes towards peers with disabilities than those in schools that did not include such students. But having a relative with a disability did not have a significant influence
Knowledge	[28]	TATDP	Participants: University students (582 from Medical School, 224 from School of Nursing) Setting: Ege University, Turkey	People who have knowledge about the attitudes towards the disabled in advance will have a better attitude.
	[22]	ATDP-form O	Participants: 587 undergraduate nursing students Setting: Three cities in Turkey	Prior knowledge has a positive impact on creativity, consciousness and development attitude
	[26]	CAMI	Participants: 62 primary care nurses Setting: three major healthcare centers in Brunei	Increase in knowledge level decreases social restrictiveness (negative) attitude
	[37]	The disability questionnaire	Participants:142 employers Setting:Colorado Springs, United States	Employer attitudes was not related to their knowledge about what constitutes ADA (Americans with Disabilities Act)
Profession	[30]	ATDP (Form B)	Participants: 634 college students and 234 healthcare professionals Setting: Tel Aviv University, Israel	X-ray technicians have lesser positive attitudes toward the person with disability than occupational therapists, nurses, family doctors and physical therapists.
	[41]	The Teacher Integration Attitudes Questionnaire	Participants: Teachers of physical education (56) and music education (54) Setting: University of Kansas, United States	Music education teachers held significantly less favorable attitudes towards children with emotional and behavioral disorders; Physical education teachers held significantly less favorable attitudes about socialization of children with orthopedic handicaps
Religion	[29]	CATCHs; MAS	Participants:200 high school and 144 university students Setting: Nijmegen, Netherlands	Religion does not influence the attitude on the disabled
	[42]	A picture-ranking interview of specific physical disabilities	Participants: 54 children with craniofacial anomalies and 68 healthy children Setting: Negros, Philippines	Religions’ beliefs are very significant for comprehending attitudes toward disabled groups
	[30]	ATDP (Form B)	Participants: 634 college students and 234 healthcare professionals Setting: Tel Aviv University, Israel	Religion does not influence the attitude on the disabled
Income	^[10]^10]	ATTID	Participants: 1605 adults Setting: Québec, Canada	Attitudes are generally not associated with income
Self-esteem	[29]	CATCHs; MAS	Participants:200 high school and 144 university students Setting: Nijmegen, Netherlands	For behavior and positive affect index, the higher the participants’ self-esteem, the more positive attitude was toward deaf and blind peers, but not toward paralyzed and intellectually disabled peers; for cognition and negative affect index, self-esteem affects attitudes toward all the disabled, except the paralyzed peers.

*ATDP* The Attitudes toward Disabled People, *CATCHs* The Chedoke-McMaster Attitudes Toward Children with Handicaps, *ATTID* The Attitudes Toward Intellectual Disability, *MAS* Multidimensional Attitudes Scale toward Persons with Disabilities, *ADS* The Attitudes to Disability Scale, *CLAS-MR* the Community Living Attitudes Scale—Mental Retardation Form, *CAMI* The Community Attitudes Towards Mental Illness Scale, *MRAI-R* The Mental Retardation Attitude Inventory-Revised, *GNAT* A Go/No-go Association Task, *DSDS* The Disability Social Distance Scale, *SADP* Scale of Attitudes towards Disabled Persons, *IM4Q* Independent Monitoring for Quality, *ID* Intellectual Disabilities, *ADA* The Americans with Disabilities Act, *TATDP* Turkish Attitudes towards Disabled Person Scale, *IDD* Intellectual and Developmental Disability

#### Demographic factors

The most commonly investigated demographic factors that associated to the attitude provider are gender, age, and education, while the impacts of other related factors such as income and religion were addressed in very few studies.

- *Gender*

A total of eight studies examined gender as an influential factor [10, 19, 20, 28–32]. Among them, significant differences were found in attitude between men and women in six studies [10, 19, 20, 28, 29, 31], while two other studies reported that there is no relationship between gender and attitudes toward people with disabilities [30, 32]. Particularly, within these six studies, five studies found that males view persons with disabilities more negatively than females [19, 20, 28, 29, 31]. However, Morin et al [10] indicated that man could have more negative attitudes than women regarding the discomfort index, whereas women had more negative attitudes regarding the knowledge of capacity and rights index than men. This may also demonstrate that scores of female tend to be more positive on behavior rather than cognition.

b) *Age*

A total of 11 studies [10, 14, 19–23, 29, 31–33] examined the association between age and public attitudes with nine of them [10, 14, 20–23, 29, 31, 32] reporting significant differences between younger and older people in the score of the attitudes. Nevertheless, disparity exists in these studies, among which four studies [21, 23, 29, 32] found that older people hold more positive attitudes than younger people toward the disabled, whereas the remaining five others [10, 14, 20, 22, 31] reported the opposite results.

c) *Education*

While two studies [19, 32] suggested a lack of evidence of an association between education level and their attitudes, three others [10, 20, 33] found that lower education level appeared to be associated with negative attitude towards disabled people.

d) *Job and income factors*

Two studies [30, 41] reported the impact of different professions on attitudes toward disabilities. One study [41] suggested that music teachers are less likely to respond favorably to children with emotional and behavioral disorders, while physical education teachers often perceive children with orthopedic defects less favorably. The other study [30] revealed that X-ray technicians have less positive attitudes toward the people with disability than occupational therapists, nurses, family, doctors and physical therapists. However, few study estimated the influence of income level to the attitudes toward the disabled. It seems that personal income level has no relationship with attitudes toward people with disabilities [10].

e) *Religion*

Three papers [29, 30, 42] had explored the relationship between attitudes and religion beliefs. One paper [42] stated that religions’ beliefs are very significant for comprehending attitudes toward disabled groups, and two others [29, 30] found that religion did not affect the attitude on the disabled groups.

#### Exposure to the disabled

- *Contact to the disabled*

Eleven studies investigated the relationship between attitude provider’s personal contact with the disabled people and attitude scores. Although these studies produced mixed results, the most consistent finding was a positive relationship between attitudes and more contacts with persons with disabilities.

i. Frequency/Quantity of the contacts. Four studies [10, 19, 34, 35] demonstrate that higher frequency of contact was associated with more favorable attitude toward people with disabilities, however, two studies [14, 36] found that higher levels of exposure could actually engender more negative attitudes toward people with disabilities.
ii. Quality of the contacts. The nature of the contact that people experienced was notably and distinctly related to their attitudes towards the disabled, as reported by two [36, 39] studies investigating this area. To specify, a higher quality of contact is predictive of a stronger positive attitude, which in turn predicts a lower repulsive attitude [36].

b) *Familiarity of the disabled*

Many studies found that ever contact with persons with disabilities through that knowledge about the policy act family, friends, social life, or elsewhere, does have a significant impact on their attitudes. Five studies [20, 24, 25, 29, 40] reported that contacting with a disabled family members, schoolmates, friends or colleagues was significantly associated with more favorable attitudes. Yet, two other studies [22, 33] found no relationship between these variables. Another study [23] surprisingly found that this association existed in the attitudes towards physical disabilities but was not apparent towards intellectually disabled persons.

c) *Knowledge about the disabled*

People’s knowledge about the disabled was also investigated in several studies. Three of them reported that people who have higher knowledge level would have better attitude toward people with disabilities [22, 28] and minimize negative attitudes [26], however, one study [37] found no relationship between attitudes and their knowledge of the policy act regarding disabled people.

#### Personality and cognitive factors

Only one study [29] investigated personal self-esteem’s impact on their attitudes. Regarding behavior and positive affection, the study reported that the higher the self-esteem status, the more positive their attitude was toward peers with hearing and visual impairments, but this was not the case for paralyzed and intellectually disabled peers; however, for cognition and negative affect items, self-esteem affected attitudes toward all the disabled peer groups, except the paralyzed peers.

### Factors related to the disabled people

Relationships between the variables related to the disabled and public’s attitudes toward them across eight studies are demonstrated in Table 3.

**Table 3:** Variables related to people with disabilities

Factors	Ref.	Instruments	Study population and setting	Results
Severity of conditions	[10]	ATTID	Participants:1605randomly selected adults Setting: Québec, Canada	Compared with higher functional intellectual disability, public attitudes toward people with lower functional tend to be more negative
	[18]	ADS	Participants: 1853 people with physical disability Setting: Guangzhou, China	Significantly negative correlation between the severity of disability and attitude towards disability
Type of disability	[29]	CATCHs, MAS	Participants:200 high school and 144 university students Setting: Nijmegen, Netherlands	Regarding the behavior and positive affect, respondents had more positive attitudes toward deaf, blind and paralyzed persons than toward intellectually disabled persons. Regarding the cognition and negative affect, respondents had more positive attitudes toward deaf and blind persons than toward paralyzed and intellectually disabled persons.
	[42]	The Teacher Integration Attitudes Questionnaire	Participants: Teachers of physical education (56) and music education (54) Setting: University of Kansas, United States	Children with emotional and behavioral disorders are considered less favorable by music education teachers, whereas children with orthopaedic disabilities are considered less favorable by teachers of physical education.
	[38]	Students’ Attitudes toward People with a Disability Scale	Participants:406 secondary school students Setting: Hong Kong	Compared with people with physical, visual or hearing impairment, students had poorer attitudes toward people with intellectual impairment and ex-mentally ill.
	[35]	A picture-ranking interview of specific physical disabilities	Participants: 54 children with craniofacial anomalies and 68 healthy children Setting: Negros, Philippines	(1) Girls show lower preference for obesity and higher preference for the arm-hand deformity. Boys, however, are more positive toward those in wheelchairs and less positive toward arm (2) Children with facial abnormalities have lower preferences than other physical disabilities.
	[36]	SADP	Participants: 338 Chinese students in three Hong Kong secondary schools Setting: Hong Kong	Chinese students have higher ratings for physically disabled people than those with emotional disturbances or mental retardation
	[30]	ATDP-Form B	Participants: 634 college students, and 234 healthcare professionals Setting: Tel Aviv University, Israel	The attitudes toward ill persons were more negative than attitudes toward injured persons, but reactions to the specific individuals presented in the vignettes were not affected by their being ill or injured.
Gender	[30]	ATDP-Form B	Participants: 634 college students, and 234 healthcare professionals Setting: Tel Aviv University, Israel	(1) Among students, gender of the disabled was unrelated to attitudes toward them (2) Among professionals, their attitudes toward male patients were more negative than toward female patients

*ATDP* The Attitudes toward Disabled People, *CATCHs* The Chedoke-McMaster Attitudes Toward Children with Handicaps, *ATTID* The Attitudes Toward Intellectual Disability, *MAS* Multidimensional Attitudes Scale toward Persons with Disabilities, *ADS* The Attitude to Disability Scale, *SADP* Scale of Attitudes towards Disabled Persons

#### Severity of the disability

Two studies [10, 18] estimated the extent to which the severity of disability on public attitude, which reported a significant negative correlation between the level of severity and attitudes toward them.

#### Type of disability

There are six [29, 30, 34, 38, 41, 42] studies examined type of disability as an influential factor of attitudes. Specifically, one research [29] found that regarding the behavioral and positive affective aspects of attitudes, respondents have more positive attitudes toward the deaf, paralyzed, and blind than intellectually disabled people. In the same study, regarding the cognitive and negative affective aspects of attitudes, both the paralyzed and the intellectually disabled were regarded with a less positive attitude than the blind and deaf. The other study [30] demonstrated that healthcare professionals showed lesser positive attitudes toward disabled people caused by illness than by injury, but these attitude differences usually do not show up during social contacts. Furthermore, there was greater public acceptance of people with physical disabilities, compared to those with mood disorders and mental retardation [34]. When it comes to children particularly, evidence shows that children depicted with facial anomalies received lower preference, compared with other visible physical disabilities [42].

#### Gender

One study [30] found that attitudes toward female patients were more positive than those toward male patients, although this result may not be consistent when responders are students.

### Factors related to society

Only one study [27] reported relationships between media and public’s attitudes toward the disabled, which showed that when controlling for gender, and contact, people who viewed the humorous media had significantly more positive attitudes to the disabled than people who did not view it. The result is demonstrated in Table 4.

**Table 4:** Factors related to the society

Factors	Ref.	Instruments	Study population and setting	Results
Media	[27]	ATDP	Participants: 133 undergraduate students majoring in business Setting: university in the southeastern, United States	Controlling for age, gender, and exposure to people with disabilities, people who watched humorous video were more positive about people with disabilities than those who didn’t.

*ATDP* The Attitudes toward Disabled People

## Discussion

### Key findings and implications

Public attitude is crucial to the disabled people with regard to their daily lives, social participation, and their integration into the community. The present systematic review of 27 studies was performed to identify different aspects of factors that may influence public’s attitude toward people with a disability. Three categories of factors were found to be associated with the public, which are related to the attitude holder/provider, disabled people, and society respectively. The key findings from this study suggested that, among a variety of factors under each category, the most recognized ones would be the knowledge (familiarity) of the disabled and the contact with the disabled, since they have been studied most frequently and have been proved to conclude more consistent and significant association with the public attitudes.

The findings from this review also indicated that gender was linked to attitudes, with men have a more negative attitude toward the person with disability than women [10, 19, 20, 28, 29, 31]. This is likely due to the nature of women’s work. To specify, with comparison to men, women are more likely to choose human service professions and therefore have more opportunities in contacting with person with disabilities, which could lead to more positive attitudes toward the disabled. Besides, maternal feelings and cultural differences in the society may account for the higher attitude scores [28]. Of note, it seems that gender only influences certain components of attitudes toward the person with disability. For instance, De Laat et al. [29] found that females had more negative attitudes on the cognition and negative affection, but not on behavior and positive affection. The authors supposed the reason under this may be that girls act more aggressively regardless of their beliefs and knowledge, while boys tend to act more in line with their beliefs [29]. This difference between male and female therapists or professionals could have implications for healthcare outcomes as well as attitudes towards the disabled and the decision to work with them.

Among all discussed factors, the finding concerning the relationship between “contact to the disabled” and the attitudes is the most-frequently discussed one. The manifestation of contact include the time length, frequency and the quality of contact with disabled people, or having friends, family members and colleagues who are disabled (“Familiarity”). The majority of studies [10, 19, 21, 23, 24, 28, 29, 34–40] have found that contacting with people with disabilities could lead to more positive attitudes towards them. This finding may because more contact could help to reduce fear and anxiety, and create a more balanced and realistic perspective about people with disability regarding to their functional capacity and ability [10, 20, 21]. Evidence shows that people who come into contact with the disabled would consider themselves more valuable in the social life and thus would be less likely to approach the disabled with rejection [28]. However, it is worth noting that without controlling demographics variables and the quality of the contact, the attitude toward disabled people would be negative. To illustrate, when the contact quality is not specifically considered, greater exposure may unexpectedly lead to uncomfortable or unpleasant feelings, and people may associate these bad experiences with the disabled people themselves [36]. Thus, it is important to consider the quality and quantity of the contact together rather than mere quantity alone in order to promote the attitudes. Although there are few studies [22, 33] found no significant difference between attitude and contact, the reasons under that is explainable (i.e., the lack of planned relationships between students and disabled people, or the low frequency of such relationships). Therefore, we believe that under certain contact conditions, advance knowledge about the disability may be necessary in order to improve the attitude towards disability. The reduction of anxiety between groups and the creation of an environment that could not only reduces prejudice, but also promote positive interactions in a more enjoyable way, suggest a continuous cycle of benefits.

We found that almost all of the studies indicated a positive relationship between the education level and attitudes. People with higher levels of education might be more liberal, open, and understanding of people with disabilities and the related issues, which would let them have a better attitude towards people with disabilities [43]. This tends to support the consideration that active interactive education could consider as a means to enhance public awareness and acceptance of people with disabilities [44]. Therefore, cooperation with disabled people can become an important part of education for the public in the future. More importantly, developing and implementing disability-specific and high quality education curricula as a part of healthcare providers’ professional program to enhance their attitude towards the disabled needs to be considered by the decision-makers.

Our results showed a lack of consistency on some factors. To illustrate, the findings regarding the relationship between the age of the respondent and their attitudes are not consistent. Some studies suggested that younger people hold more negative attitudes toward person with disabilities than those of the older [20, 21, 23, 29, 32], while opposite findings were also reported in several other studies [10, 14, 22, 31]. This may because the participants’ characteristics of each study [20, 29] are not homogeneous, and more likely, the results were not controlled by other potential explanatory factors, e.g., knowledge about the disabled, or the contact with them [21]. For example, some health professionals were offered a series of programs which aims to eliminate stereotypes about disabled people, then their previous experience in dealing with the disabled and their views on the disabled may explain the difference in their attitudes compared others who weren’t trained [22, 31]. Previous research have concluded that knowledgeable about people with disabilities and related issues could lead people to have more favorable attitudes toward the disabled [43]. The knowledge of the disabled might also inform selection to the medical profession, or specific training programs within healthcare medicine. Therefore, these results could provide an insight that we should pay more attention to the education of young people about issues related to disabilities, and increase the duration of their contact with the disabled people appropriately as well. Thus, in designing future evaluations, researchers should bear in mind that studies that random assignment of participants to groups can provide reliable evidence about the effects of age.

With regard to other potential factors (such as religion, income and humorous media), religion and income were found to have no effect on people’s attitudes towards disabled people, while humorous media have a positive impact. Evidence suggest that humor may have a normalizing effect during an abnormal situation [45], and its impact on reducing anxiety have been well documented in many fields in several studies [46–48]. Another possible explanation could be that humorous introductions provide a non-threatening and anxiety-reducing means of incorporating constructive images of disability into mainstream culture to communicate information about disability [49]. Thus, it is recommended to use a comprehensive humorous approach to provide information on persons with disabilities, which could become an effective way to change negative attitudes [50].

In additional to the factors discussed above, it is found that public attitude is also dependent upon the individual factor of the disabled, e.g., type of disability. Several studies [29, 34, 38] found that, among all the disability types, attitudes toward persons with intellectually/mentally disabled are less positive than those with other types. On the other hand, people tend to have less positive attitude toward the individuals with more visible physical impairment [39, 42]. A possible reason behind this finding is based on the idea that persons with intellectually disability could exhibit unpredictable behavior and therefore pose a threat [10, 29]. Moreover, people tend to have less positive attitude toward the individuals with more visible physical impairment [42]. To specify, public always hold less negative attitudes to the blind and deaf compared to the paralyzed and the intellectually disabled [29], which maybe because a paralysis and intellectual disability are more visible than deafness and blindness, and people with facial anomalies would be less favorably perceived compared to those with other physical disabilities.

### Measurement employed

In this review we found that the included studies used a great variety of scales to evaluate the attitudes toward the disabled people, and the ATDP (Attitude Towards Disabled People) [51] was the most used one by nine studies [19, 22, 23, 27, 28, 30, 32, 33, 40]. This instrument was developed in 1960, and has three forms: form O is the original form with 20 items; form A and form B, both with 30 items, are improved versions of form O. It is a research-validated instrument for measuring generalized attitudes toward persons with general disabilities. ATDP has sound psychometric properties which have demonstrated reliability and content and construct validity evidence [51, 52]. A further included 2 studies [23, 34] used SADP (Scale of Attitudes Towards Disabled Persons) [53], which was developed in 1981 to provide an alternative to the ATDP Form-O to measure the general population’s attitudes towards disabilities in general. SADP has also been found to be reliable and content-validated [53]. These two scales-ATDP and SADP, are both appropriate for research regarding general participants and not limited to contact with one type of disability. Although they are widely used and extensively researched in the contemporary studies, these scales couldn’t be used to reflect the impact of difference between persons with various disabilities. Other instruments used in else included studies measure specific dimensions of attitudes toward specific disabilities (e.g., ATTID, The Attitudes Toward Intellectual Disability [54]) within specific contexts (e.g., CLAS-MR, the Community Living Attitudes Scale-Mental Retardation [55]), or in specific age groups (e.g., CATCHs, The Chedoke-McMaster Attitudes Towards Children with Handicaps [56]). Whether studies have reported the psychometric properties of the chosen measure of attitudes to indicate their validation was not part of our eligibility criteria of inclusion. To note, a latest study in 2020 [57] mentioned another popular tool to measure the attitudes towards the disabled in various aspects- the Attitudes to Disability Scale (ADS) developed by the WHOQOL Group [58], which was proved to be useful and reliable for studying the whole society in general. Future studies could consider employing this measurement as well.

### Knowledge gaps

Among the included studies, there is a lack of evidence about a progression of or improvement in attitudes occurred as time passed. To date, long-term prospective studies that evaluate changes in attitude across time are needed, in order to better understand the best way to cultivate positive attitudes from education to practice. As previously noted, most of the literatures reviewed in this study were based on student samples, which rendering their findings unrepresentative of the general public. In addition, the diversity of the assessment instrument used may obfuscate the results to be applied generally. Future studies assessing attitudes toward people with disabilities need to use more rigorous design that includes validated measurements so a future meta-analysis may be feasible.

According to our search results, compared to factors related to the attitude provider, social factors and factors related to the disabled themselves were less studied. This indicates the need for more in-depth studies. There is limited evidence of the relationship between income level and the attitudes in the current studies. Future studies could situate the current findings within a broader context such as links to bodies of research on stigma, empathy/fatigue in healthcare providers. Besides, as the reviewed literature originated from different countries, possible cultural differences should be taken into account when establishing such a guide for the future studies in this area.

## Strengths and limitation of this review

### Strength

We sought to provide as comprehensive an overview of attitudes toward the disabled as possible, rather than focusing on a specific angle (e.g., specified type of disability) in this area. We have not identified any previous study that attempted to systematically review the attitudes of the general public toward people with all kinds of disabilities, which makes our review meaningful and important. The data related to the factors, setting, sample, instruments employed, and the results were extracted for each study, which could provide a clear awareness and understanding of the feature of the included studies, and provide the insight of the current knowledge gaps to inform further studies in this area. The findings of this study have important implications for the education and training of the future health professionals.

### Limitation

Only English records were retrieved for this review, which means that insights from papers written in other languages could have been missed, and the language and publication bias may be present. Another limitation is the decision to exclude records from the qualitative study and grey literature, which may decreased the breadth of insights generated. However, as our aim is to find out those studies who concluded quantitative relationships between different measureable parameters by using statistical models and analyses methods, qualitative studies and grey literatures may not give us the best evidence to find out the association. In addition, cc, thus, studies prior to that year would have been missed. And we are unable to meta analyze the results given the heterogeneous nature of the included data in terms of study design, populations studied, and instruments used.

## Conclusion

This systematic review identified three categories related to the factors associated with attitudes toward the disabilities, which indicate an association with attitudes towards the disabled: factors related to the attitude provider, disability themselves, and the society. Among all of the factors, provider’s knowledge of the disability and their contact with the disabled would be the most recognized ones that influence attitude towards people with disabilities. ATDP scale was the most frequently used scale to measure attitudes in the included studies. Additional focus may be necessary for future study to randomly assign participants (student and non-student sample) to provide more representative and comprehensive evidence. Future research should aim to establish a practical guide based on these factors proved to be influential, which would provide not only the health care professionals but the general lay public with strategies for interacting with individuals with disabilities, and inform effective decisions to the stakeholders in order to build a more inclusive society.

## Data Availability

Data sharing is not applicable to this article as no datasets were generated or analyzed during the current study.

## Abbreviations

ADA: The Americans with Disabilities Act
ADS: The Attitudes to Disability Scale
ATDP: The Attitudes toward Disabled People
CATCHs: The Chedoke-McMaster Attitudes Toward Children with Handicaps
ATTID: The Attitudes Toward Intellectual Disability
CAMI: The Community Attitudes Towards Mental Illness Scale
CLAS-MR: The Community Living Attitudes Scale—Mental Retardation Form
DSDS: The Disability Social Distance Scale
GNAT: A Go/No-go Association Task
ID: Intellectual Disabilities
IDD: Intellectual and Developmental Disability
IM4Q: Independent Monitoring for Quality
MAS: Multidimensional Attitudes Scale toward Persons with Disabilities
MRAI-R: The Mental Retardation Attitude Inventory-Revised
SADP: Scale of Attitudes towards Disabled Persons
TATDP: Turkish Attitudes towards Disabled Person Scale
